# The Local Structure and Metal-Insulator Transition in a Ba_3_Nb_5−x_Ti_x_O_15_ System

**DOI:** 10.3390/ma15134402

**Published:** 2022-06-22

**Authors:** G. M. Pugliese, F. G. Capone, L. Tortora, F. Stramaglia, L. Simonelli, C. Marini, Y. Kondoh, T. Kajita, T. Katsufuji, T. Mizokawa, N. L. Saini

**Affiliations:** 1 Department of Physics, Sapienza University of Rome, P. le Aldo Moro 2, 00185 Roma, Italy; gianmarco.pugliese@uniroma1.it (G.M.P.); federico.capone@synchrotron-soleil.fr (F.G.C.); lorenzo.tortora@uniroma1.it (L.T.); fstramaglia@gmail.com (F.S.); 2 CELLS—ALBA Synchrotron Radiation Facility, Carrer de la Llum 2-26, Cerdanyola del Valles, 08290 Barcelona, Spain; lsimonelli@cells.es (L.S.); cmarini@cells.es (C.M.); 3 Department of Applied Physics, Waseda University, Tokyo 169-8555, Japan; y-kondoh@akane.waseda.jp (Y.K.); tomomasa@fuji.waseda.jp (T.K.); katsuf@waseda.jp (T.K.); mizokawa@waseda.jp (T.M.)

**Keywords:** TTB niobates, metal-insulator transition, local structure, X-ray absorption spectroscopy

## Abstract

The local structure of the filled tetragonal tungsten bronze (TTB) niobate Ba${}_{3}$Nb${}_{5-x}$Ti${}_{x}$O${}_{15}$ (*x* = 0, 0.1, 0.7, 1.0), showing a metal-insulator transition with Ti substitution, has been studied by Nb K-edge extended X-ray absorption fine structure (EXAFS) measurements as a function of temperature. The Ti substitution has been found to have a substantial effect on the local structure, that remains largely temperature independent in the studied temperature range of 80–400 K. The Nb-O bonds distribution shows an increased octahedral distortion induced by Ti substitution, while Nb-Ba distances are marginally affected. The Nb-O bonds are stiffer in the Ti substituted samples, which is revealed by the temperature dependent mean square relative displacements (MSRDs). Furthermore, there is an overall increase in the configurational disorder while the system with Nb 4*d* electrons turns insulating. The results underline a clear relationship between the local structure and the electronic transport properties suggesting that the metal-insulator transition and possible thermoelectric properties of TTB structured niobates can be tuned by disorder.

## 1. Introduction

The variety of electronic and structural properties of transition metal oxides (TMOs) [1] have made research on this class of solids increasingly challenging and fascinating. Among them, research interest largely accelerated on the TMOs with perovskite structure after the discovery of high T${}_{c}$ superconductivity in La${}_{2-x}$Ba${}_{x}$CuO${}_{4}$ [2] while other TMOs such as tetragonal tungsten bronze (TTB) oxides [3,4] were comparatively overlooked, although they are among perovskites derivatives. Besides superconductivity, perovskites also show a wide range of electronic and magnetic properties, ranging from metallic to insulating behavior. For example, BaTiO${}_{3}$ and PbTiO${}_{3}$ perovskites are known for their ferroelectric properties driven by Ti 3*d*-O 2*p* hybridization while Sr${}_{1-x}$La${}_{x}$TiO${}_{3}$ is found to result in a large thermoelectric performance [5,6].

**Figure 1:**
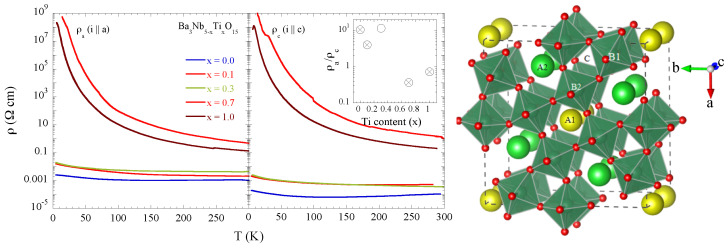
Temperature dependent resistivity of Ba${}_{3}$Nb${}_{5-x}$Ti${}_{x}$O${}_{15}$ single crystal samples for different Ti concentration measured with electrical current parallel to the *a*-axis (**left**) and the *c*-axis (**middle**) of single crystal samples. The system with *x* = 0.0 is an anisotropic metal, that turns gradually to an isotropic insulator for *x* = 0.7 and 1.0. The resistivity anisotropy has been shown as a function of Ti substitution in the inset (middle) with the crossed circles indicating the samples used for the local structure study. The crystal structure of TTB, drawn using VESTA is shown (**right**) [20].

Recently, TTB oxides have been studied for a range of functional properties including their dielectric, ferroelectric to relaxor behavior [3,4]. The special properties of TTB oxides are due to flexibility in composition, design and freedom of structural manipulation. Indeed, TTB oxides are characterized by a general formula A1${}_{2}$A2${}_{4}$B1${}_{2}$B2${}_{8}$C${}_{4}$O${}_{30}$, containing interlinked perovskite-like units forming triangular, square, and pentagonal interstitial spaces (Figure 1). The triangular space (C) is generally vacant with square and pentagonal spaces (A1 and A2) occupied in a “filled” TTB while vacancies at A1 and A2 in the structure makes the TTB “unfilled”. Among others, “unfilled” TTB niobates, (Sr,Ba)${}_{5}$Nb${}_{10}$O${}_{30}$, i.e., (Sr,Ba)Nb${}_{2}$O${}_{6}$ have been investigated for their complex crystal structure favouring low thermal conductivity [7,8,9,10,11]. However, low electrical conductivity limits their thermoelectric performance determined by the thermoelectric figure of merit $ZT=S^{2}T/\kappa \rho$, where *S* is the Seebeck coefficient, *T* is the absolute temperature, ρ is the electrical resistivity and κ is the thermal conductivity [12]. They have also been studied for their dielectric response showing a crossover from ferroelectric to relaxor behavior with Sr substitution [13,14]. The “filled” TTB niobates, with a random occupation of the A1 and A2 structural sites, have also been studied for their dielectric response [15]. Besides, Ba${}_{3-x}$Sr${}_{x}$Nb${}_{5}$O${}_{15}$ shows metal to insulator transition by partial substitution of Sr, assigned to an increased atomic disorder at Nb${}_{2}$O${}_{12}$ octahedral sites [16,17,18]. Indeed, Ba${}_{3}$Nb${}_{5}$O${}_{15}$ is an anisotropic metal that turns to an isotropic insulator by the partial Sr substitution [18]. However, a complete understanding of the metal-insulator transition in this class of materials is still under discussion [16,17,18].

The filled TTB niobates have potential in the field of ferroelectrics and thermoelectrics in which the properties can be controlled by selective substitutions to manipulate their flexible structure. For example, Ba${}_{3}$Nb${}_{4}$TiO${}_{15}$, with partial substitution of Ti at the Nb site, is another filled TTB niobate having the potential for controllable thermoelectric response [19]. Ba${}_{3}$Nb${}_{5-x}$Ti${}_{x}$O${}_{15}$ shows metal-insulator transition (Figure 1) with partial Ti substitution for Nb, very similar to the one found in Ba${}_{3-x}$Sr${}_{x}$Nb${}_{5}$O${}_{15}$ [16,17,18]. Resistivity measurements, shown in Figure 1, point out a clear evolution of the electric transport properties as a function of Ti concentration from an anisotropic metal for *x* = 0 to an isotropic insulator at *x* = 0.7 and 1.0. Since the Ti valence is +4, the *x* = 0.7 system would be an exotic insulator with the partially filled Nb 4*d* bands while the *x* = 1.0 system is just a band insulator with the empty Nb 4*d* bands. In this work, we have studied the local structure of Ba${}_{3}$Nb${}_{5-x}$Ti${}_{x}$O${}_{15}$ (*x* = 0.0, 0.1, 0.7, 1.0) by Nb K-edge extended X-ray absorption fine structure (EXAFS) measurements in the temperature range of 80–400 K to explore possible cause of the observed metal-insulator transition in this filled niobate system. The results show a clear correlation between the electric transport and the local structure providing a direct evidence of structural disorder induced metal-insulator transition in the filled TTB niobates.

## 2. Materials and Methods

The single crystal samples of Ba${}_{3}$Nb${}_{5-x}$Ti${}_{x}$O${}_{15}$ were grown by the floating-zone method as described for Ba${}_{3-x}$Sr${}_{x}$Nb${}_{5}$O${}_{15}$ system [18]. More details on the preparation and characterization together with the optical conductivity of Ba${}_{3}$Nb${}_{5-x}$Ti${}_{x}$O${}_{15}$ will be reported elsewhere [21]. Resistivity measurements (Figure 1) were performed using a four-probe method with the applied current in two high symmetry directions, i.e., i‖a and i‖c axis of the single crystal samples. Temperature dependent Nb K-edge (∼18,985 eV) X-ray absorption measurements were carried out at the CLÆSS beamline of ALBA synchrotron in Cerdanyola del Valles (Barcelona) [22]. The synchrotron beam emitted by a multipole wiggler source was vertically collimated using water cooled mirrors and monochromatized by a double crystal Si(311) monochromator followed by focussing on the samples using dual toroid mirrors. Powdered samples of Ba${}_{3}$Nb${}_{5-x}$Ti${}_{x}$O${}_{15}$ were mixed uniformly in an organic matrix (cellulose) and pressed into pellets of 5 mm of diameters with thicknesses of the pellets optimized to obtain a unitary jump at the Nb K-edge X-ray absorption measurements in the transmission mode. The samples were cooled by a continuous flow liquid nitrogen cryostat with a temperature control accuracy of ±1 K. The samples were measured simultaneously with a reference using three ionization chambers. Data reproducibility and high signal-to-noise ratio were ensured by several absorption scan acquisitions. Standard procedure based on polynomial spline functions [23,24] was used to extract the EXAFS oscillations. The absorption spectra of the samples were acquired sequentially in the same experimental conditions and the EXAFS oscillations were extracted using the same spline function approach.

## 3. Results and Discussion

**Figure 2:**
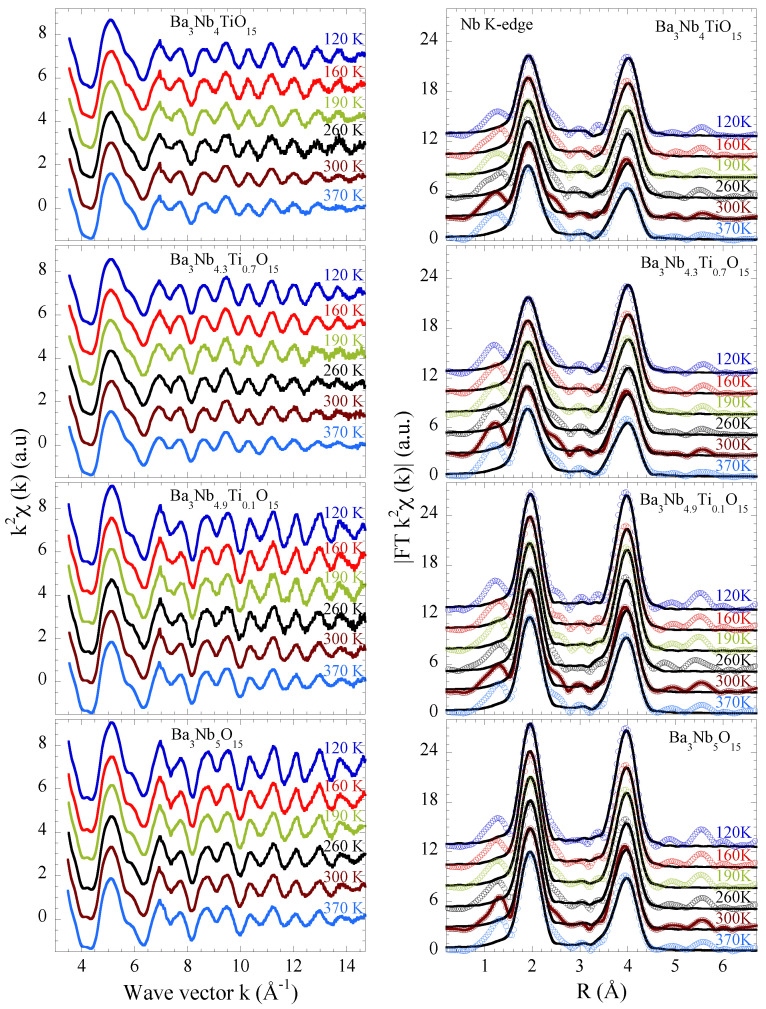
Nb K-edge EXAFS oscillations of Ba${}_{3}$Nb${}_{5-x}$Ti${}_{x}$O${}_{15}$ samples (*x* = 0.0, 0.1, 0.7, 1.0) for several temperatures (left panels). The oscillations are multiplied by $k^{2}$ and vertically shifted for a better visualization. The corresponding Fourier transforms (FTs) magnitudes are shown in the right panels. The EXAFS model fits to the FTs are also shown as black solid lines.

Here we focus on the Nb K-edge EXAFS of Ba${}_{3}$Nb${}_{5-x}$Ti${}_{x}$O${}_{15}$ (*x* = 0.0, 0.1, 0.7, 1.0) to have direct access to the local structure around the niobium atoms. The EXAFS oscillations, extracted from the Nb K-edge X-ray absorption spectra, are shown at several temperatures in Figure 2 (left). The EXAFS are shown multiplied by $k^{2}$ to amplify the oscillations at higher *k*-values. In addition to the thermal damping, the EXAFS oscillations also show a substantial change with Ti substitution indicating higher configurational disorder for the substituted samples. The effect of Ti substitution on the local structure can be viewed in real space by the Fourier transforms (FTs) of the EXAFS oscillations providing partial atomic distribution function. The FT magnitudes are displayed in the right panels of Figure 2. The FTs displayed in the figure are performed in the k-range of 3.0–15 Å${}^{-1}$ using a Gaussian window function.

The Ba${}_{3}$Nb${}_{5-x}$Ti${}_{x}$O${}_{15}$ shown in Figure 1 has a typical tetragonal tungsten bronze structure (a = 12.598 Å, b = 3.9774 Å, space group P4/*mbm)* in which niobium atoms occupy the centers of the octahedra denoted here as B1 and B2. Square and pentagonal interstitial spaces are occupied by barium atoms denoted as A1 and A2, whereas there are vacancies in the triangular ones (C). In the structure of the filled TTB niobate with chemical formula Ba${}_{3}$Nb${}_{5}$O${}_{15}$ (*x* = 0), the NbO${}_{6}$ octahedra centered on the B1 sites are more distorted with Nb-O distances ranging from ∼1.957 Å to ∼2.068 Å compared to the B2 sites centered octahedra showing a Jahn-Teller like distortion with four Nb-O distances at ∼1.965 Å and two at ∼1.9887 Å [25]. Therefore, on average there is a distribution of Nb-O distances in the studied system. The next neighbors of Nb are Ba atoms at ∼3.4 Å (B2 sites) and at ∼3.65 Å (B1 sites) followed by the next Nb atoms. In the FTs shown in Figure 2 (right), the two main peaks between R ∼ 1.5–4.5 Å takes into account the contributions of the Nb-O bondlenghts (the first peak) and Nb-Ba distances as well as the Nb-Nb (∼3.74 Å) and Nb-Ba (∼3.97 Å) (the second peak). On the other hand, diffraction data on Ba${}_{3}$Nb${}_{4}$TiO${}_{15}$ (*x* = 1.0) reveal a slightly different tetragonal structure with space group P4*bm* with lattice parameters a = 12.53 Å and c = 4.01 Å [26,27].

In order to obtain the local structural parameters we have modeled the EXAFS oscillations in the single scattering approximation following the general equation [23,24]:

(1) $$\chi \left(k\right)=\sum_{i}\frac{N_{i}S_{0}^{2}}{kR_{i}^{2}}f_{i}\left(k,R_{i}\right)e^{-\frac{2R_{i}}{\lambda }}e^{-2k^{2}\sigma^{2}}sin\left[2kR_{i}+\delta_{i}\left(k\right)\right]$$

where $N_{i}$ is the number of neighboring atoms at distance $R_{i}$ from Nb, $S_{0}^{2}$ is the EXAFS amplitude reduction factor due to many-body effects related to inelastic excitation channels as shake-up and shake-off excitations, $\delta_{i}$ is the phase shift, λ is the photoelectron mean free path, $f_{i}\left(k,R_{i}\right)$ is the backscattering amplitude and $\sigma_{i}^{2}$ is the EXAFS Debye-Waller factor representing the mean square relative displacement of the photoabsorber-backscatter pairs. The starting parameters for the EXAFS model fits were taken from the XRD data on Ba${}_{3}$Nb${}_{15}$O${}_{15}$ sample [25] with the shells including Nb-O distances, Nb-Ba distances and Nb-Nb distances. The EXCURVE 9.725 code was used (with calculated backscattering amplitudes, photoelectron mean free paths and phaseshift functions) for the non linear least square fits of the EXAFS oscillations [28]. The two Nb${}_{2}$O${}_{12}$ octahedra are characterized by a distribution of Nb-O distances; however, they were found to merge in two Nb-O distances in the EXAFS fits. Therefore, for simplicity, the distribution has been treated as if characterized by two different distances having about two third (shorter) and one third (longer) probability in the final analysis. Interatomic distances ($R_{i}$) and the corresponding MSRD parameters ($\sigma_{i}^{2}$) were allowed to vary in the fit procedure while the coordination numbers $N_{i}$ were kept fixed. The amplitude reduction factor ($S_{0}^{2}=0.8$ for Nb-O and $S_{0}^{2}=1.0$ for Nb-Ba and Nb-Nb shells) and photoelectron energy zero ($E_{0}=2.945$ eV) were kept fixed after trials on different scans and reference samples. The fitting *k*-range and *R*-range were 3.0–15 Å${}^{-1}$ and 1.5–4.5 Å respectively, thus the number of independent data points for the fits is 2ΔkΔR/π∼23 with the number of fits parameters being 12. The goodness of fit, shown by black solid lines in Figure 2 (right) is determined by the *R*-factor defined as:

(2) $$R=\frac{\sum_{i=1}^{N}{\left|\chi_{th}\left(r_{i}\right)-\chi_{exp}\left(r_{i}\right)\right|}^{2}}{\sum_{i=1}^{N}{\left|\chi_{exp}\left(r_{i}\right)\right|}^{2}}$$

where *N* is the number of data points, $\chi_{th}\left(r_{i}\right)$ and $\chi_{exp}\left(r_{i}\right)$ the theoretical and experimental EXAFS signal, respectively. The *R*-factors were found to be ∼4±1 for the samples with *x* = 0.0 and 0.1 and ∼7±2 for *x* = 0.7 and 1.0 showing the fit to be slightly worse for the latter.

**Figure 3:**
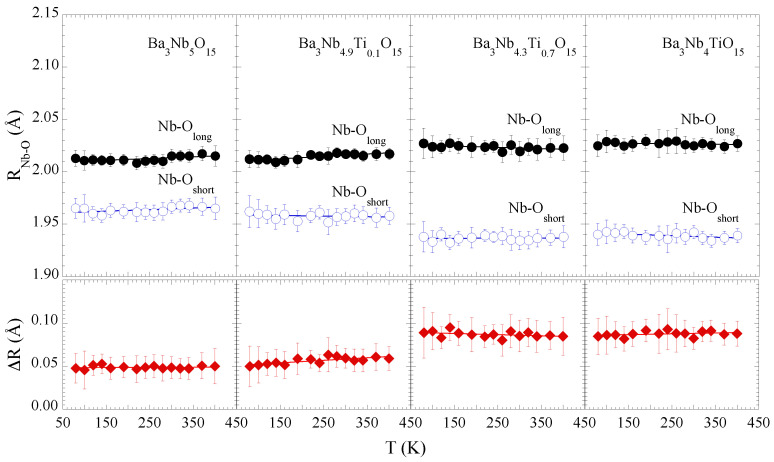
Nb-O distances for Ba${}_{3}$Nb${}_{5-x}$Ti${}_{x}$O${}_{15}$ (*x* = 0.0, 0.1, 0.7, 1.0) are shown as a function of temperature in upper panels. Empty and filled circles represent short and long Nb-O bondlengths. The Nb-O bondlengths (ΔR) separations are also plotted in lower panels (filled diamonds). The solid lines are linear fit to guide the eyes. The error bars represent maximum uncertainty calculated by the analysis of different EXAFS scans.

Figure 3 shows the Nb-O distances as a function of temperature for different Ti substitutions. Apparently, there is a marginal effect of temperature on the local structure of the Nb${}_{2}$O${}_{12}$ octahedra, seems to be the case for all the samples, albeit the Ti substitution affects substantially the local Nb-O bonds distribution. This observation is evident from the separation between the longer and shorter Nb-O bondlengths (lower panels). The mean Nb-O separation is ∼0.05 Å for *x* = 0.0, 0.1, that increases up to ∼0.09 Å for *x* = 0.7, 1.0, i.e., on an average the NbO${}_{6}$ octahedral distortions tend to increase with Ti concentration (*x* = 0.7, 1.0). Since the conduction band consists of Nb 4d and O 2p states, a change in the octahedral configuration is likely to affect the orbitals hybridization and hence the transport properties of the system [25]. We will come back to discuss this later.

The two Nb-Ba bond distances are found to be ∼3.53 ± 0.01 Å and ∼3.65 ± 0.01 Å for *x* = 0.0, 0.1 samples and show an elongation due to Ti substitution. However, the separation between the two Nb-Ba distances remains unaffected for all four samples (not shown), with the Nb-Ba distances being ∼3.56 ± 0.01 Å and ∼3.69 ± 0.01 Å for *x* = 0.7, 1.0 samples. This is indicative that Ti substitution also affects the B1 and B2 sites (Figure 1) in the filled TTB niobate. Similarly, the Nb-Nb bond distance, ∼3.79 ± 0.02 Å found to show a usual thermal expansion, albeit slightly longer than the one reported in diffraction studies (∼3.74 Å [25]). The Nb-Nb distance also shows a small elongation with Ti substitution.

The EXAFS Debye-Waller factors $\sigma_{i}^{2}$ measuring the mean square relative displacements, i.e., the distance-distance correlation function, of the absorber-backscatter pair of atoms [23,24], provide further information on the local bond dynamics. The temperature dependence of $\sigma_{i}^{2}$(T) can be described by the Einstein Model given by the following equation [29,30]:

(3) $$\sigma_{i}^{2}=\sigma_{0}^{2}+\frac{\hbar^{2}}{2k_{B}\mu \Theta_{E}}coth\left(\frac{\Theta_{E}}{2T}\right)$$

where $k_{B}$ is the Boltzmann constant, μ is the reduced mass of the atomic pairs. $\sigma_{0}^{2}$ is an offset related with the overall static disorder along a specific bondlength and $\Theta_{E}$ is the Einstein temperature. Temperature dependent EXAFS permits to determine the Einstein temperature giving direct access to the bond properties since it is strictly related with the bond stiffness $k=\mu \omega_{E}^{2}$ ($\Theta_{E}$ = $\omega_{E}$/$k_{B}$, with $\omega_{E}$ being the Einstein frequency).

**Figure 4:**
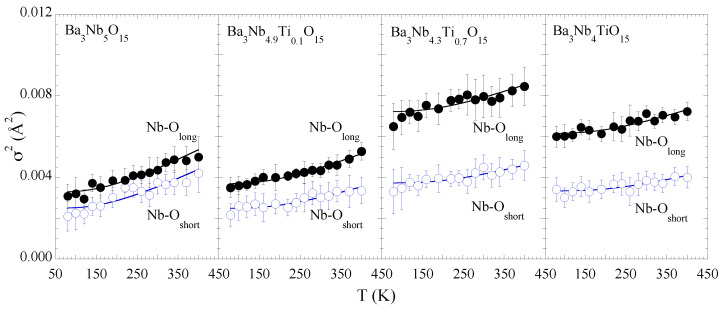
Temperature dependence of the mean-square relative displacement parameter ($\sigma^{2}$) of Nb-O distances for Ba${}_{3}$Nb${}_{5-x}$Ti${}_{x}$O${}_{15}$ for *x* = 0.0, 0.1, 0.7, 1.0. Empty and filled circles are used to represent the short and long Nb-O bonds. The solid lines are the Einstein model fits (see text).

Figure 4 shows the $\sigma^{2}$(T) of the two Nb-O distances as a function of temperature. The Einstein temperatures for the two distances show a gradual increase with Ti concentration indicating that the two bonds tend to get stiffer by the partial substitution. Besides, there is an overall increase in the configurational disorder with Ti substitution. The $\sigma_{0}^{2}$ does show an increase with the Ti substitution and the largest configurational disorder appears in the sample with *x* = 0.7. In general, configurational disorder weakens bondlengths unless there is some kind of texturing due to the partial substitution. Therefore, the local stiffening of the Nb-O bonds together with the increased configurational disorder could be due to Ti-texturing. The Einstein temperatures for the Nb-O bonds together with the $\sigma_{0}^{2}$, indicating Nb-O configurational disorder, are shown in Table 1.

**Figure 5:**
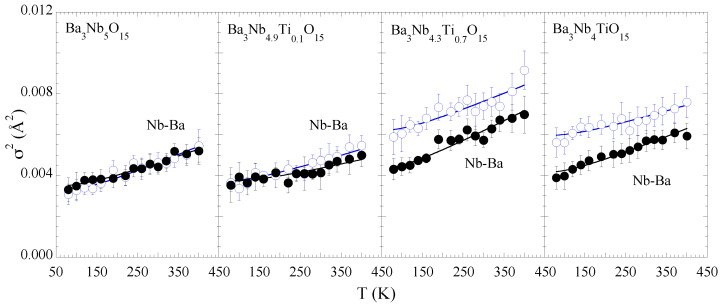
Temperature dependence of the mean-square relative displacement parameter ($\sigma^{2}$) of Nb-Ba distances for Ba${}_{3}$Nb${}_{5-x}$Ti${}_{x}$O${}_{15}$ (*x* = 0.0, 0.1, 0.7, 1.0). Empty and filled circles represent the short and long Nb-Ba bonds. Nb-Ba bonds. The solid lines are the best fit results of the Einstein model.

**Figure 6:**
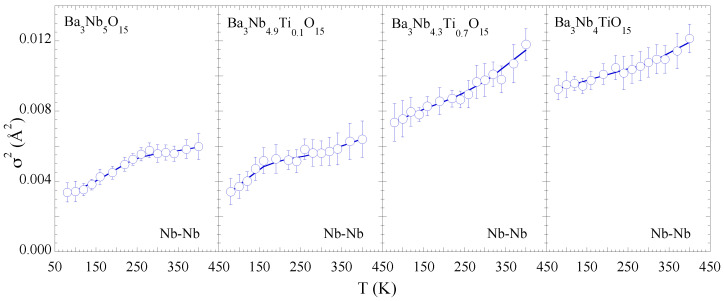
Temperature dependence of the mean-square relative displacement parameter ($\sigma^{2}$) of Nb-Nb distance. The solid lines are guide to the eyes showing a small temperature dependent anomaly for *x* = 0 and 0.1 samples.

Figure 5 shows the MSRD of the two Nb-Ba distances. For the consistency we have used the Einstein model to describe the temperature dependence of the MSRD to determine the bond characteristics. The $\Theta_{E}$ values for Nb-Ba hardly show any systematic change with Ti substitution except a tendency of increased configurational disorder. This may be a likely effect of the Nb site disorder due to substitution affecting the Nb-Ba correlations. The Einstein temperatures for the Nb-Ba bonds are also included in Table 1. On the other hand, the temperature dependence of the Nb-Nb MSRDs tends to show an anomalous change for the samples with *x* = 0.0 and 0.1, appearing respectively at ∼300 K and ∼200 K while the anomalous change seems suppressed for the *x* = 0.7, 1.0 samples (Figure 6). We speculate that the small anomaly is related with some kind of spontaneous atomic order in the complex structure reflected in the Nb-Nb correlations. Nevertheless, the overall configurational disorder, i.e., $\sigma_{0}^{2}$, increases for both Nb-Ba and Nb-Nb distances.

Coming back to the main results of this work, it is clear that the local structural parameters remain largely temperature independent except the small anomalies in Nb-Nb correlations for Ba${}_{3}$Nb${}_{5}$O${}_{15}$ and Ba${}_{3}$Nb${}_{4.9}$Ti${}_{0.1}$O${}_{15}$. Instead, the local structure shows a clear evolution with a partial Ti substitution. The main effects are; (i) increased Nb-O bonds distribution indicating octahedral distortions; (ii) increased Nb-O bonds stiffness and an overall configurational disorder; (iii) a marginal effect on the Nb-Ba network except an overall increased configurational disorder. It has been discussed earlier [25], that the nearly metallic Ba${}_{3}$Nb${}_{5}$O${}_{15}$ is highly sensitive to impurities/defects that can affect the electronic properties of these systems. Indeed, special performances of TTB structured compounds are believed to be intrinsically related with their complex compositional flexibilities and tunable crystal structures. In the TTB structured niobates, the electronic properties are mainly driven by the overlap of the Nb 4d and O 2p orbitals; therefore, the evolution observed in local structural parameters of the octahedra (Figure 3 and Figure 4) with partial substitution of Ti for Nb should affect the transport properties of the system.

Similar to Ba${}_{3-x}$Sr${}_{x}$Nb${}_{5}$O${}_{15}$ [17,18], Ti substitution in Ba${}_{3}$Nb${}_{5-x}$Ti${}_{x}$O${}_{15}$ largely affects the transport properties, as evident in Figure 1. Indeed, the metallic Ba${}_{3}$Nb${}_{5}$O${}_{15}$, showing anisotropic resistivity, turns insulating with partial Ti substitution. The local structure measurements clearly show an increased overall local structural disorder, evident from both Nb-O distance distribution (Figure 3) and the increased configurational disorder, apparent from the Nb-O MSRDs (Figure 4). Therefore, the results show an intrinsic correlation between the local structure of NbO${}_{6}$ octahedral network and the metal-insulator transition in the TTB niobates. Interestingly, the sample with *x* = 0.7 is more insulating than the one with *x* = 1.0, and also characterized by higher NbO${}_{6}$ disorder. This observation makes a further distinction between the exotic insulator and the band insulator. It is likely that Ba${}_{3}$Nb${}_{4}$TiO${}_{15}$ to be more homogenous phase (empty *d* band) with respect to Ba${}_{3}$Nb${}_{4.3}$Ti${}_{0.7}$O${}_{15}$ (partially filled *d* bands), thus favouring the electrical conductivity.

**Figure 7:**
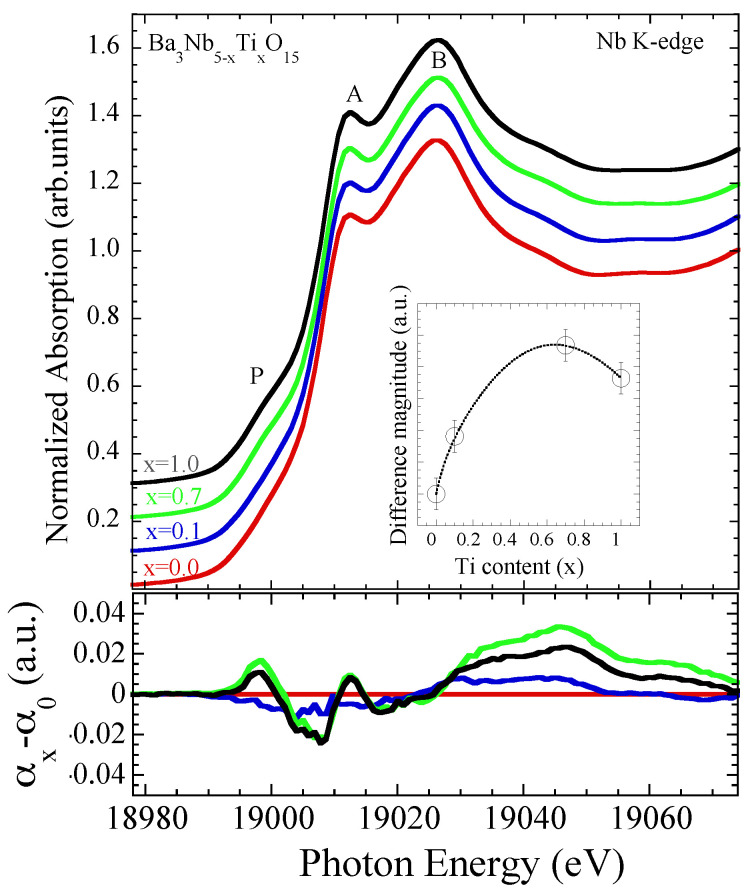
Normalized Nb K-edge XANES spectra of Ba${}_{3}$Nb${}_{5-x}$Ti${}_{x}$O${}_{15}$ (*x* = 0.0, 0.1, 0.7, 1.0) at 300 K (upper). The spectra are artificially shifted for a better visualization. The XANES differences with respect to the one for *x* = 0.0 are also shown (lower). The inset shows integrated absolute spectral XANES difference as a function of Ti substitution. The error bars in the intensity represent maximum error estimated by analyzing five different absorption scans for each sample.

The EXAFS results reveal substantial change in the local structure with Ti substitution including increased Nb${}_{2}$O${}_{12}$ octahedral distortions and an overall configurational disorder in the system. The X-ray absorption near edge structure (XANES) region of the Nb K-edge absorption spectra, a probe of the local geometry and the valence electronic states [23,24] can provide further information on the system. Figure 7 shows normalized Nb K-edge XANES spectra of Ba${}_{3}$Nb${}_{5-x}$Ti${}_{x}$O${}_{15}$ (*x* = 0.0, 0.1, 0.7, 1.0) at room temperature (300 K), characterized by two peak features A and B together with a pre-peak shoulder P. The feature A, well separated from the broad feature B, is mainly due to the dipole allowed transition from Nb 1*s* to the unoccupied Nb 5*p* states while the structure B is a multiple scattering feature involving admixed states from different near neighbor orbitals in the continuum thus carrying useful information on the local geometry. The pre-peak feature P is expected to be due to quadrupole transition from the Nb 1*s* to the unoccupied Nb 4*d* states admixed with the *p* orbitals and considered to be a direct probe of the Nb coordination symmetry [31,32]. Indeed, the intensity of the feature P is a direct measure of the distortion in the coordination symmetry around the Nb atom, i.e., Nb${}_{2}$O${}_{12}$ octahedral distortions.

The XANES features show small changes in their spectral intensity with Ti substitution. Although a detailed analysis with an appropriate theoretical model is required for the complete understanding of different XANES features, a qualitative evolution can be obtained by looking at the spectral differences. The changes with Ti substitution can be seen from the differences between the XANES spectra. We have plotted the XANES differences with respect to the spectrum for *x* = 0.0 sample in Figure 7 (lower). The maximum difference ranges between 2–4% with a clear change in the shoulder feature P as well as continuum beyond the feature B. Indeed, the shoulder feature P intensity increases with Ti substitution as the case of the spectral weight beyond the peak feature B. The increased intensity of feature P is consistent with the increased Nb octahedral distortions observed by EXAFS. The increasing spectral difference reflects changes in the local geometry as well as the valence electronic states. This can be seen further from the integrated absolute spectral difference plotted as a function of Ti substitution in the inset of Figure 7. Incidentally, the largest difference appears for the sample with *x* = 0.7, consistent with the EXAFS findings, thus making a clear distinction between the exotic insulator with partially filled Nb 4*d* bands for *x* = 0.7 and the band insulator with empty Nb 4*d* bands for *x* = 1.0.

## 4. Conclusions

In summary, we have studied the local structure of Ba${}_{3}$Nb${}_{5-x}$Ti${}_{x}$O${}_{15}$ (*x* = 0.0, 0.1, 0.7, 1.0) using temperature dependent Nb K-edge extended X-ray absorption fine structure (EXAFS) measurements. On an average, the local structure of Ba${}_{3}$Nb${}_{5-x}$Ti${}_{x}$O${}_{15}$ remains temperature independent, however, the Ti substitution largely affects the local configuration of the Nb-O network. The Debye-Waller factors, providing direct information on the bond characteristics, reveal increased stiffness of the Nb-O bonds suggesting increased Nb 4d-O 2p hybridization. Besides the bond stiffness, the Nb-O network suffers an overall configurational disorder by Ti substitution. The results suggest that the metal-insulator transition in Ba${}_{3}$Nb${}_{5-x}$Ti${}_{x}$O${}_{15}$ from an anisotropic metal (*x* = 0.0 and 0.1) to an isotropic insulator with partially filled Nb 4*d* bands (*x* = 0.7) is mainly driven by the local octahedral distortions and configurational disorder in the system. The XANES spectra show changes consistent with the EXAFS results. Incidentally, the similar metal-insulator transition is known to occur in Ba${}_{3-x}$Sr${}_{x}$Nb${}_{5}$O${}_{15}$ with Sr substitution, assigned to be caused by structural disorder. Further studies on the thermal conductivity and Seebeck coefficient would be helpful for the optimization of the thermoelectric properties of these materials through manipulation of the local disorder by substitution.

## Figures and Tables

**Table 1:** The local structure parameters of Ba${}_{3}$Nb${}_{5-x}$Ti${}_{x}$O${}_{15}$ determined by Nb K-edge EXAFS. The distances ($R_{i}$) are shown at room temperature (300 K) while the Einstein temperatures ($\theta_{E}$) are determined by the temperature dependence of $\sigma_{i}^{2}$. The temperature-independent terms ($\sigma_{0}^{2}$), related with configurational disorder, are also included in the table.

	Nb-O${}_{\mathrm{short}}$			Nb-O${}_{\mathrm{long}}$			Nb-Ba${}_{\mathrm{short}}$			Nb-Ba${}_{\mathrm{long}}$		
x	**R (Å)**	$\theta_{E}$ **(Å)**	$\sigma_{0}^{2}$ **(Å${}^{2}$)**	**R (Å)**	$\theta_{E}$ **(Å)**	$\sigma_{0}^{2}$ **(Å${}^{2}$)**	**R (Å)**	$\theta_{E}$ **(Å)**	$\sigma_{0}^{2}$ **(Å${}^{2}$)**	**R (Å)**	$\theta_{E}$ **(Å)**	$\sigma_{0}^{2}$ **(Å${}^{2}$)**
0.0	1.97	573 ± 53	0.0001(5)	2.02	556 ± 47	0.0002(5)	3.52	316 ± 24	0.0018(3)	3.65	349 ± 31	0.0022(3)
0.1	1.96	696 ± 88	0.0001(2)	2.02	625 ± 46	0.0009(3)	3.53	364 ± 31	0.0025(2)	3.66	420 ± 72	0.0026(4)
0.7	1.93	741 ± 98	0.0014(5)	2.02	650 ± 95	0.0046(7)	3.57	317 ± 34	0.0048(4)	3.69	301 ± 28	0.0028(3)
1.0	1.94	772 ± 99	0.0011(5)	2.02	676 ± 62	0.0036(4)	3.56	371 ± 49	0.0048(4)	3.69	321 ± 28	0.0028(4)

## Data Availability

The data presented in this study are available on reasonable request from the corresponding author.
